# Effects of light environment during growth on the expression of cone opsin genes and behavioral spectral sensitivities in guppies (*Poecilia reticulata*)

**DOI:** 10.1186/s12862-016-0679-z

**Published:** 2016-05-18

**Authors:** Yusuke Sakai, Hajime Ohtsuki, Satoshi Kasagi, Shoji Kawamura, Masakado Kawata

**Affiliations:** Department of Ecology and Evolutionary Biology, Graduate School of Life Sciences, Tohoku University, 980-8578 Sendai, Japan; Department of Integrated Biosciences, Graduate School of Frontier Sciences, University of Tokyo, 277-8562 Kashiwa, Japan

**Keywords:** Opsin, Color vision, Light environment, Sexual signals, Sensory drive

## Abstract

**Background:**

The visual system is important for animals for mate choice, food acquisition, and predator avoidance. Animals possessing a visual system can sense particular wavelengths of light emanating from objects and their surroundings and perceive their environments by processing information contained in these visual perceptions of light. Visual perception in individuals varies with the absorption spectra of visual pigments and the expression levels of opsin genes, which may be altered according to the light environments. However, which light environments and the mechanism by which they change opsin expression profiles and whether these changes in opsin gene expression can affect light sensitivities are largely unknown. This study determined whether the light environment during growth induced plastic changes in opsin gene expression and behavioral sensitivity to particular wavelengths of light in guppies (*Poecilia reticulata*).

**Results:**

Individuals grown under orange light exhibited a higher expression of long wavelength-sensitive (LWS) opsin genes and a higher sensitivity to 600-nm light than those grown under green light. In addition, we confirmed that variations in the expression levels of LWS opsin genes were related to the behavioral sensitivities to long wavelengths of light.

**Conclusions:**

The light environment during the growth stage alters the expression levels of LWS opsin genes and behavioral sensitivities to long wavelengths of light in guppies. The plastically enhanced sensitivity to background light due to changes in opsin gene expression can enhance the detection and visibility of predators and foods, thereby affecting survival. Moreover, changes in sensitivities to orange light may lead to changes in the discrimination of orange/red colors of male guppies and might alter female preferences for male color patterns.

**Electronic supplementary material:**

The online version of this article (doi:10.1186/s12862-016-0679-z) contains supplementary material, which is available to authorized users.

## Background

Habitats can have significant influences on the evolution of sexual signals [1, 2]. Accordingly, various environmental factors, such as food abundance, predation pressure, parasites, and other biotic/abiotic factors affect the expression of sexual signals and preferences [3, 4]. Sensory drive models for sexual selection can be used to investigate the effects of various environmental factors on signal perception and subsequent sexual selection [5], and environmental conditions can act as transmission filters for sexual signals, leading to sexual selection that favors more detectable sexual trait variants in particular environments [4–7].

Because a broad range of animals use coloration as a sexual signal, the visual system has become one of the most actively studied sensory drive systems. Perception and detection for color signals are determined by several different components [8]. One important component is the light sensitivity of individuals for a given spectral condition of the environment. The mechanism and degree to which individuals can detect light of a certain range of wavelengths depend on the given spectral sensitivity of the individuals. For instance, the retinas of cichlid fishes inhabiting relatively blue-shifted environments are more sensitive to the blue wavelength of light than those from relatively red-shifted environments [9], and these could be associated with sensory drive through female choice [9, 10]. During the initial stage, such biases in sensitivity to light arise due to variations in the properties of photoreceptor cell response [11]. Molecular mechanisms of visual perception have been well studied at the level of peripheral processing [12–15], and it is accepted that visual pigments comprise opsin proteins and chromophores (vitamin A derivatives) that transduce light signals in the environment into electrochemical signals in the neural system [16]. Moreover, opsin proteins play important roles in spectral sensitivities of visual pigments. Vertebrates possess five types of visual opsins: 1) rhodopsin 1 (RH1) responsible for dim light perception; 2) RH2, the color green; 3) short wavelength-sensitive type 1 (SWS1), ultraviolet (UV) blue; 4) short wavelength-sensitive type 2 (SWS2), blue; and 5) the middle-to-long wavelength-sensitive (M/LWS)-type opsins, red–green [14]. Specific amino acid substitutions in opsin proteins generate shifts in photoreceptor sensitivity to light wavelengths [14, 17].

In addition to amino acid substitutions, the differential gene expressions of opsins have crucial effects on spectral sensitivities of visual pigments [18]. Differential expressions of opsin genes in different light environments have been reported in several species of fishes. For example, African cichlids in Lake Victoria show variations in the expression of the SWS2B opsin gene across taxa, which are correlated with the spectral composition of the environment [19]. In bluefin killifish (*Lucania goodei*), individuals inhabiting springs of high water clarity and high transmission of UV and blue wavelengths exhibit higher expression of SWS1 and SWS2B opsin genes, whereas individuals inhabiting swamps where the water color is red shifted with low transmission of UV and blue wavelengths exhibit higher expression of RH2 and LWS [20].

Several studies have demonstrated plastic changes in opsin gene expression according to light environments. In the bluefin killifish, differential expression of opsin genes observed across populations could be experimentally re-created. Individuals raised in tea-stained treatments had higher expression levels of RH2 and LWS, whereas those from clear-water treatments had higher expression levels of SWS1 and SWS2B [21]. Similar plastic differentiations were observed in African cichlids from Lake Malawi, and the lab-reared individuals which raised in a UV-minus light environment expressed opsin genes differently from wild-caught individuals [22]. These studies suggest that light environments could alter the expression levels of opsin genes, which consequently affect spectral sensitivity to the light environments.

The guppy (*Poecilia reticulata*) has been an important model organism for studies of ecology, behavior, and evolution, particularly for studies of sexual selection. Male guppies have highly polymorphic body color patterns, and female preference for some components of these male color patterns varies among populations in response to environmental factors [23–25]. Archer et al. (1987) [26] and Archer and Lythgoe (1990) [27] showed that spectral sensitivities of photoreceptor cells to middle and long wavelength ranges varied between individual guppies. Subsequently, Endler (1992) [5] suggested that variations in visual properties contribute to differences in female preferences for male color patterns. Recent molecular genetic studies also revealed that guppies carry nine opsin genes, including an ultraviolet-sensitive gene (*SWS1*), two subtypes of blue-sensitive genes (*SWS2-A* and *SWS2-B*), two subtypes of green-sensitive RH2 genes (*RH2-1* and *RH2-2*) and, remarkably, four subtypes of red-sensitive LWS genes (*LWS-1*, *LWS-2*, *LWS-3*, and *LWS-4*) [28–31]. For LWS genes, the nomenclature of the first three genes follows [32] and that of the last follows [31]. In addition to spectral variations among loci, studies showed allelic spectral variations in LWS-1 opsin [33] in feral populations in Okinawa. Moreover, an amino acid substitution in LWS-1 opsin protein sequences was found to correspond with residue 180 in human M/LWS opsins (180 Ala and 180 Ser) [31–33], which is one of five key positions that influence wavelength sensitivity [34, 35].

Moreover, expression levels of certain opsin genes reportedly vary significantly among guppies and have been associated with differences in sex and age of individuals [36]. Sandkam et al. [37] examined differences in the opsin gene expression levels among populations experiencing different predation pressures (high and low predation), and found that individuals inhabiting low-predation environments expressed higher levels of LWS opsin genes than those inhabiting high-predation environments. Female guppies show consistent preferences for carotenoid-based red and orange spots in males [24, 25], which reflect light at wavelengths >500 nm that are mainly absorbed by LWS opsins [38, 39]. Sandkam et al. [37] therefore suggested that habitat-specific visual characteristics via differential expression levels of LWS opsins may result in differences in female preferences for red and orange spots. However, it remains unknown how and which environmental factors change the expression profiles of opsin expression, and whether or not these differences affect spectral sensitivities that may cause differences in color vision and subsequent female preferences.

In the present study, we determined whether light environment during development alters the expression of opsin genes, and whether these changes affect spectral sensitivities to specific light wavelengths. To this end, we assessed the effects of light environment during the juvenile stage on the levels of opsin expression in the adult stage of the guppy. Previous studies of bluefin killifish (*L. goodei*) [21] and the cichlids in Lake Malawi [22] showed that the expression levels of opsins could change according to the different environmental conditions. Hence, adult guppies may adapt to light environments by altering spectral sensitivity during growth stages. In the present study, we correlated the expression levels of nine opsin genes with spectral sensitivities to specific light wavelengths according to optomotor behavioral responses.

## Results

### Behavioral responses to long wavelengths of light

**Fig. 1:**
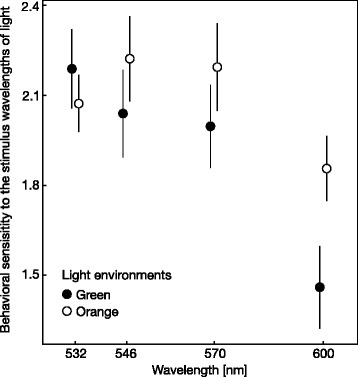
Behavioral sensitivities to the stimulus wavelengths of light of individuals grown under green light (*N* = 5) and those grown under orange light (*N* = 5) at various wavelengths. The behavioral sensitivity was calculated as the negative logarithm of threshold detectable light intensities (μmol/m^2^/s) according to optomotor responses (see Methods section). *Circles* indicate the means ± standard errors of the mean (SEM)

We were able to measure the behavioral sensitivity to long wavelengths of light for ten individuals, five under green light (two females and three males) and five under orange light (three females and two males), because 10 of 15 individuals readily followed the moving stripes, whereas five did not and remained at one side of the cylinder, even under the strongest light intensity. The behavioral sensitivities varied between the four tested wavelengths, with sensitivity to 600-nm light being lower than that to the shorter wavelengths (Table 1; Fig. 1). Moreover, the significant interaction between orange light during growth and the 600-nm wavelength light indicated that individuals grown under orange light were significantly more sensitive to 600-nm light than those grown under green light (Table 1; Fig. 1).

**Table 1:** Generalized linear mixed model (GLMM) of behavioral sensitivities to the four stimulus wavelengths of light with negative logarithms of threshold detectable light intensity values as a response variable and individual identifications as a random effect

Explanatory variables	*df*	Estimate	S. E. M	*t*	*P*
Light environments (Orange)	22.65	−0.116	0.214	14.47	0.5934
Wavelength (546 nm)	22.65	−0.149	0.161	−0.54	0.3578
Wavelength (570 nm)	64	−0.192	0.161	−0.93	0.2385
Wavelength (600 nm)	64	−0.728	0.161	−4.52	<0.0001
Env. (Orange) : Wave. (546 nm)	64	0.299	0.228	1.31	0.1941
Env. (Orange) : Wave. (570 nm)	64	0.313	0.228	1.38	0.1738
Env. (Orange) : Wave. (600 nm)	64	0.511	0.228	2.25	0.0281

### Relative expression levels of cone opsin genes

**Fig. 2:**
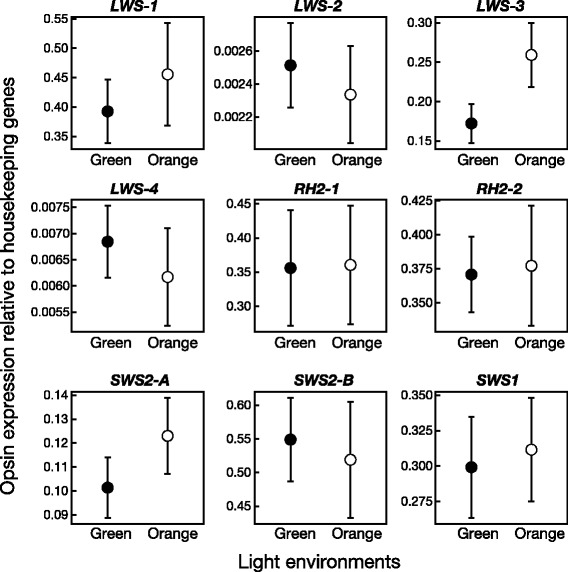
The mean (± standard errors of the mean (SEM)) expression values of cone opsin genes relative to housekeeping genes according to separate quantitative polymerase chain reaction (qPCR) experiments for individuals grown under green light (*Green*, *N* = 8) and those under orange light (*Orange*, *N* = 6). Gene expression values of cone opsins were normalized to the geometric mean value of the expression of housekeeping genes

The expression levels of nine opsin genes could be obtained in 14 individuals (eight individuals under green light [four females and four males] and six under orange light [five females and one male]), since one individual under orange light had died before quantitative polymerase chain reaction (qPCR) experiments. Figure 2 shows the expression values for each opsin gene relative to the geometric mean expression of the three housekeeping genes. In these experiments, *LWS-3* expression was significantly greater in individuals under orange light than in those under green light (Table 2; Fig. 2). However, no differences were found in the expression levels of the other eight opsin genes between individuals under different light environments (Table 2).

**Table 2:** Effects of light environment during growth on the relative expression levels of nine cone opsin genes; generalized linear mixed models (GLMMs) were generated for nine opsin genes using the opsin expression values relative to house keeping genes as response variables

Gene	*df*	*χ* ^2^	*P*
*LWS-1*	1	2.27	0.132
*LWS-2*	1	0.00	0.990
*LWS-3*	1	4.24	0.039
*LWS-4*	1	1.22	0.269
*RH2-1*	1	0.00	0.969
*RH2-2*	1	0.02	0.882
*SWS2-A*	1	1.69	0.194
*SWS2-B*	1	0.13	0.718
*SWS1*	1	0.06	0.810

### The relationship between behavioral sensitivity to long wavelengths of light and the expression profiles of the opsin gene

**Fig. 3:**
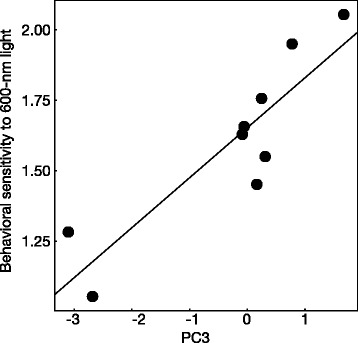
The relationship between the principle component 3 (PC3) and the behavioral sensitivity to 600-nm light. The gene expressions of long-wavelength-sensitive (*LWS*)*-1* and -*3* positively contribute to PC3 values (see text and Additional file 1: Table S4). The values of behavioral sensitivity were the negative logarithm of threshold detectable light intensities

Principal component analysis (PCA) showed that the first three PCA axes accounted for 79.3 % of the variation in the expression levels of all opsin genes. More specifically, PC1 showed a negative correlation with the proportional expressions of *SWS2-A*, *LWS-4*, *SWS1*, and *RH2-2*, whereas PC2 was negatively correlated with the proportional expression of *RH2-1* and positively correlated with the expression of *LWS-1* and *SWS2B* (Additional file 1: Table S1). PC3 was positively correlated with the proportional expression of *LWS-1* and *LWS-3* and negatively correlated with the proportional expression of *LWS-2*, *LWS-4*, *RH2-2*, and *SWS2-B* (Additional file 1: Table S1). Subsequent generalized linear model (GLM) analyses showed that among the three PC axes, the light environmental conditions during growth had significant effects on PC3 scores (Table 3) and the mean PC3 scores were higher under orange light than under green light. In addition, the increase in the PC3 scores and concomitant increase in the expression of *LWS-1* and *LWS-3* led to significant increases in behavioral sensitivity to 600-nm light (Table 4; Fig. 3). In contrast, the PC1 and PC2 scores had no effects on behavioral sensitivity to the four tested wavelengths of light.

**Table 3:** Effects of light environment during growth on principle components (PCs) 1, 2, and 3; generalized linear models (GLMs) were generated using PCs as response variables and light environment during growth as a fixed effect

Principal components	*df*	*χ* ^*2*^	*P*
PC1	1	0.31	0.5798
PC2	1	0.00	0.9539
PC3	1	4.06	0.0439

**Table 4:** The effects of PC1, PC2, and PC3 on behavioral sensitivities to the four stimulus wavelengths of light; GLMs were generated for each wavelength using behavioral sensitivities to the stimulus wavelengths of light as response variables

Explanatory variables	Wavelength	*df*	*χ* ^*2*^	*P*
PC1	532 nm	1	0.56	0.4537
	546 nm	1	0.19	0.6663
	570 nm	1	0.19	0.6590
	600 nm	1	0.00	0.9786
PC2	532 nm	1	1.77	0.3256
	546 nm	1	0.97	0.6655
	570 nm	1	3.76	0.0524
	600 nm	1	0.04	0.8411
PC3	532 nm	1	1.04	0.3073
	546 nm	1	4.94	0.0262
	570 nm	1	6.00	0.1430
	600 nm	1	25.95	<0.0001

## Discussion

In the present study, we examined the effects of light environment during growth on behavioral sensitivities to long wavelengths of light and opsin gene expression in the guppy (*P. reticulata*). In these experiments, individuals maintained under orange light during growth exhibited higher expression of a long-wavelength-sensitive opsin gene (*LWS-3*) and had higher sensitivity to orange light (600 nm) than did those maintained under green light. In previous studies of cichlids [22, 40] and killifishes [20], light environment during growth caused plastic changes in opsin gene expression; similar plastic changes occurred in guppies in the present study. The present results indicate that changes in expression levels of opsin genes lead to changes in spectral sensitivity to orange light, although factors other than opsin gene expression, i.e., optical properties of the lens [41] and connections between cone cells and neurons [42], might affect the change in the spectral sensitivity. In the present study, we did not examine the effect of sex on the changes in gene expression, since there was an insufficient number of individuals for comparison; however, this effect should be examined in future studies.

### Changes in behavioral sensitivity to long wavelengths of light and opsin gene expression

The present results showed that sensitivities to 600-nm light were remarkably lower than those to 532-, 546-, and 570-nm light. This result potentially reflects peak absorption spectra (λ_max_ values) of opsin proteins in guppy photoreceptor cells. Accordingly, 600-nm light is mainly absorbed by LWS-1 opsin (λ_max_, 562 or 571 nm). In contrast, the other three wavelengths (532, 546, and 570 nm) could be absorbed by LWS-1, LWS-2 (λ_max_, 516 nm), LWS-3 (λ_max_, 519 nm), RH2-1 (λ_max_, 516 nm), and RH2-2 (λ_max_, 476 nm) opsins (see Additional file 1: Figure S3). Our results of optomotor response were consistent with those by Endler [38], who reported decreasing sensitivities to long wavelength of light (at the range of wavelength >600 nm). These facts indicate that guppies are less sensitive to orange and red colors (long wavelengths of light) than blue, green, and yellow colors (short/middle wavelengths of light).

The intensity levels (μmol/m^2^/s) of light through the neutral density filter using the same lens density values varied between the four stimulus wavelengths, particularly under lower lens density values (Additional file 1: Figure S2), and the light intensity levels were lower under 532 and 546 nm than under 570 and 600 nm for almost all lens densities. Thus, behavioral sensitivity under 532-nm and 546-nm light might be somewhat underestimated due to an individual possibly stopping the optomotor response at a one-step higher lens density under 532/546-nm light than under 570/600-nm light. Thus, if the variation of light intensity affects the results, behavioral sensitivity was predicted to be lower under 532/546-nm light than under 570/600-nm light. However, the results showed that behavioral sensitivity was higher under 532/546-nm light than under 570/600-nm light. Thus, the variation did not affect the results. Furthermore, we directly compared the lens density values for which optomotor responses were less than 0.5 (Additional file 1: Table S2). The lens density values under which most individuals stop optomotor responses ranged from 3.0 to 3.5. There was a little variation in the light intensities among the four stimulus wavelengths for this range of lens density values; thus, variation in the light intensities did not greatly affect the results.

The present results indicate that different light environments during growth (green or orange) affected behavioral responses to 600-nm light, but not the other three wavelengths. Moreover, the light environment during growth affected the expression levels of *LWS-3*. The results of PCA showed that individuals with high PC3 values were more sensitive to 600-nm light. Positive PC3 values indicated higher expression of *LWS-1* and *LWS-3*. Hence, plastically regulated changes in *LWS-3* and potentially expression of *LWS-1* lead to changes in the behavioral sensitivities to 600-nm light. Although *LWS-3* opsin absorb little 600-nm light, individuals with high expressions of *LWS-1* and *LWS-3* showed high sensitivity to 600-nm light because the expression levels of *LWS-1* and *LWS-3* were positively correlated (Additional file 1: Figure S4); thus, the correlated increased expressions of both *LWS-1* and *LWS-3* might affect the sensitivity to long wavelengths of light. The optomotor response has been used to investigate which cone types contribute to the detection of motion, and several studies suggested that the detection of motion is mediated by one cone type only, namely the LWS opsin [40, 43, 44]. Thus, the ability of the detection of motion could be affected by plastic changes in the expression of LWS opsins.

The estimated total sum of quantum catches by nine opsins in guppies was different under the orange and green light (almost 1.8-fold higher under green light than under orange light). Thus, green light might appear brighter to guppies than orange light. There is a possibility that the difference in brightness (i.e., the total amount of light captured by opsins) affects changes in opsin expression and behavioral sensitivity. However, the present results showed that the degree of changes in expression differed for the different opsin genes so that the changes in the expression of opsin genes might be affected by the relative amount of light captured by each opsin gene rather than the total amount of light. A previous study [45] reported that guppies reared under low light conditions exhibited a weaker response to visual cues during foraging than those reared under higher light conditions. This shows that individuals grown under green light (i.e., a brighter environment) might have exhibited lower optomotor responses than those grown under orange light. However, our result indicates a higher sensitivity to orange light in guppies grown under orange light. Thus, the difference in brightness between the two light environments might not have a significant effect on behavioral sensitivities.

### Heritable and environmental effects on variations in the expression of opsins

Changes in opsin expression due to phenotypic plasticity have been reported in some species, such as bluefin killifish [21] and East African cichlids [22]. In the present study, differing light environments during growth caused plastic changes in LWS opsin gene expression levels of guppies. However, there is a possibility that individual variations in opsin expression are heritable. Endler [46] showed that behavioral sensitivity to different colors of light could be evolved under artificial selection pressures, potentially reflecting selection for genetic variations in the levels of expression of opsin. Moreover, Sandkam et al. [37] recently identified divergence of opsin expression across guppy populations in Trinidad, potentially reflecting genetic differences. Thus, further studies are required to examine the relative importance of genetic and/or environmental variation in opsin gene expression.

### Evolutionary implications of plastic changes in sensitivity to predominant wavelengths of light in habitat environments

The present results suggest that guppies grown in orange water environments had increased perceptions of orange light. Aquatic light environments for guppies vary from clear to tannin-stained. In tannin-stained water, long wavelengths of light are more effectively transmitted than short wavelengths; therefore, these aquatic environments become orange-shifted light environments [24, 38]. Under these orange-shifted aquatic environments, guppies with enhanced sensitivity to the background orange lights might more effectively detect objects that are darker than background light, such as silhouettes of predators or foods [7, 47]. Guppies inhabit shallow watersheds with spatially and seasonally varying spectral composition [38]; thus, plastic changes in sensitivity to background light environments might contribute to survival in variable light environments.

Changes in sensitivity to long wavelengths of light due to expression levels of LWS opsin genes may also alter preferences for orange spots. In Trinidad, females from orange-shifted water showed a stronger preference for males with orange and black spots [24]. For females with higher sensitivity to predominant wavelengths of orange light, the color combination of “bright” orange and “dark” black spots could be perceived as high contrast under orange-shifted light environments. Thus, enhanced sensitivity to environmental light owing to plastic changes in LWS opsin gene expression might contribute to female preference for orange and black spots of males under orange-shifted waters. However, the present study lacked a sufficiently large sample size to assess the effects of spectral sensitivities on female preferences for orange spots. Thus, further behavioral mate choice trials using individuals reared under different light conditions require investigation in future studies.

## Conclusion

The results of the current study indicate that individuals have higher sensitivities to environmental light conditions through plastic changes in LWS opsin genes, suggesting advantages for food acquisition and predator avoidance under aquatic environments characterized by longer light wavelengths. Moreover, the results provides important insights into the evolution of female preference through sensory drive, as changes in sensitivity to orange light due to the expression levels of LWS opsins may affect the perception of orange spots in male guppies.

## Methods

### Sample selection and light treatments

Twenty pregnant female guppies were collected from a long-established feral population in Gushiken (26°41ʹ46.8ʺN, 127°54′39.4″E), Okinawa Prefecture, Japan, during 2013, and were placed in a single aquarium to give birth. Fifteen juveniles were randomly chosen from the aquarium, and then randomly allocated to two 10-L tanks of different light conditions, i.e., green and orange light. These light conditions were produced using acetate filters (CL139 for green and CL105 for orange; Lee Filters) and a daylight-color light-emitting diode bulb (ECOHiLUX; Iris Ohyama). Light wavelengths ranged from 490 to 590 nm with a 530 nm respectively (Additional file 1: Figure S1A). Individuals were kept under a 12-h light/12-h dark cycle at 25 ± 1 °C and were fed daily with newly hatched brine shrimp (*Artemia salina*) and commercial flake food (Tetramin, Tetra Werke) for 6 months. Finally, eight adult individuals were placed under green light (four males and four females) and seven adult individuals under orange light (five females and two males).

To estimate photoreceptor stimulation, the number of photons absorbed by visual pigments (relative quantum catches) was calculated as follows:

$$ Q={\displaystyle \int I\left(\lambda \right)R\left(\lambda \right)d\lambda, } $$

where *I* (*λ*) represents normalized spectral irradiance and *R* (*λ*) represents opsin absorbance spectra. To calculate the spectral irradiance, sidewelling light was measured in the center of each tank using a spectrometer (USB2000, Ocean Optics) connected to a 2-m optical fiber (P-400-2-UV/VIS, Ocean Optics) fitted with a cosine corrector (CC-3-UV-S, Ocean Optics) and calibrated using a calibration light source (LS-1-CAL, Ocean Optics). Absorbance spectra of cone opsins were calculated from the average λ_max_ values for each opsin based on the equation used by Govardovskii et al. [48]. These λ_max_ values were measured for SWS1 (353 nm), SWS2-B (408 nm), SWS2-A (438 nm), RH2-1 (516 nm), RH2-2 (476 nm), Ala 180 type (A-type) LWS-1 (562 nm), Ser 180 type (S-type) LWS-1 (571 nm), LWS-2 (516 nm), LWS-3 (519 nm), and LWS-4 (ND) using in vitro reconstitution [33]. The photon flux densities (μmol/m^2^/s) of the two light environments in the range of 300 to 800 nm were very similar (0.166 μmol/m^2^/s for green light and 0.162 μmol/m^2^/s for orange light). On the other hand, the relative quantum catches for both LWS-1 opsin alleles were larger under orange light than under green light, whereas those for LWS-2, LWS-3, RH2-2, and RH2-1 opsins were greater under green light than under orange light (Additional file 1: Figure S1B). Because green light is absorbed by multiple cone opsins compared to orange light, which was largely absorbed by an LWS-1 opsin, the estimated total sum of quantum catches by nine opsins in guppies was different between orange and green lights (almost 1.8-fold higher under green light than under orange light).

### Optomotor responses

Optomotor responses refer to innate tendencies of animals to follow moving visual patterns in the absence of other stronger stimuli, and are used to determine behavioral sensitivities of different colors of light [44]. We measured behavioral sensitivities to particular light wavelength bands of individuals by observing optomotor responses. Optomotor responses were observed using an apparatus comprising of a stationary acrylic cylinder tank (diameter 12 cm) in which the tested fish could swim freely, as described by Krauss and Neumeyer (2003) [44]. The tank was concentrically surrounded by a cylinder (14 cm diameter) of 2 cm-wide white cardboard stripes with equally wide slits. The striped cylinder was placed on a rotatable acrylic disk, which was turned by a motor (US206-401 2GN18K, Oriental Motor Co.) in both directions and at various speeds. The light source was a 100-V 650-W halogen lamp, and was used to illuminate the test cylinder and the white stripes of the rotating cylinder from above. Interference filters (Band Pass Interference Filters, MELLES GRIOT) were used to obtain quasi-monochromatic light (10 nm bandwidth) and light intensities were attenuated using neutral density filters (ND filters, MELLES GRIOT). Fish behaviors were monitored from below using a video camera (HDR-CX630V, Sony) and were simultaneously observed on a monitor (KV-14MF1, Sony). A black cotton cloth and corrugated plastic board were installed around the cylinder to provide high contrast between the slits and white cardboard stripes. Optomotor responses were quantified as optomotor gains [44] using the following equation:

$$ \mathrm{Optomotor}\ \mathrm{gain} = \left(\mathrm{S}\mathrm{W}-\mathrm{S}\mathrm{A}\right)/\mathrm{N}\mathrm{R} $$

where SW and SA indicate numbers of rounds that the test fish swam per minute with, and against the direction of rotation, respectively, and NR is the number of rotations in a given direction per minute. Optomotor experiments were performed at 10 rpm and adopted a gain of 0.5 as the threshold criteria for motion detection because all fish tested exhibited gains from −0.4 to 0.4 under conditions without any moving stimuli.

To ensure complete light adaptation, experiments were initiated in the rearing aquaria at least 3 h after lights on and light-adapted individuals were transferred into the test tank and acclimatized to the cylinder under white light for 5 min. Subsequently, white light was replaced with monochromatic light, and rotation of the stripe pattern by the motor was initiated. Optomotor responses were recorded after 10 s to eliminate the effects of startle responses that many fish exhibit at the beginning of the pattern movement. Optomotor responses were then recorded for 1 min, and cylinder rotation was halted. White light was provided for 2 min before the next test, which was conducted at the same wavelength of monochromatic light but with light intensity reductions of 0.3 lens densities (D) (D = log (1/T), where T is the transmissivity) using a series of four neutral density filters. These trials were repeated with serial 0.3-D reductions of intensity, until optomotor gains were less than 0.5. Serial sessions were performed using cylinder motion in only one direction and were performed in the opposite direction on subsequent days. Downwelling light intensities of monochromatic light were measured in the middle of the acrylic cylinder tank in μmol/m^2^/s using a spectrometer (USB2000, Ocean Optics). Line plots of optomotor gains were plotted against the logarithm of light intensity (μmol/m^2^/s) for each session of a single individual. The intersection of each line plot with the threshold criterion at a gain of 0.5 was recorded, and the light intensity required to reach the threshold (threshold detectable light intensity) was interpolated. The negative logarithm of the threshold detectable light intensity was regarded as behavioral sensitivity to the stimulus wavelength. Optomotor responses of fish were measured at stimulus wavelengths of 532, 546, 570, and 600 nm, which were within the absorbance spectrum ranges of LWS opsins. The attenuation properties of light intensity of the four stimulus wavelengths against lens density values (D) are shown in Additional file 1: Figure S2, and the estimated relative quantum catches from each wavelength light are shown in Additional file 1: Figure S3. Fifteen guppies were tested under all four stimulus wavelengths, which were presented in random order using a random-number generator. The individuals were maintained in the rearing tanks under full-spectrum fluorescent lighting throughout the optomotor experiments and were kept until euthanasia prior to qPCR experiments.

### Gene expression analyses

Guppies were euthanized with an overdose of 2-phenoxyethanol after 10 h in the light phase. Sampling times corresponded with maximal expression of cone opsins at the end of the photopic day, as indicated in previous studies [49, 50]. Left and right eyes of each individual were immediately excised and separately placed into tubes containing RNAlater® Stabilization Solution (Thermo Fisher Scientific). These eye samples were subsequently homogenized in 0.5 mL of TRIZOL® solution (Thermo Fisher Scientific), and RNA was extracted using chloroform, precipitated with isopropanol, and purified using Qiagen RNeasy mini kits (Qiagen), using on-column RNase-free DNase I (Qiagen) treatment to eliminate genomic DNA contamination. Subsequently, 100 ng of isolated total RNA was transcribed into cDNA using High Capacity cDNA Reverse Transcription kits (Thermo Fisher Scientific). Real-time PCR for the opsin genes *LWS-1*, *LWS-2*, *LWS-3*, *LWS-4*, *RH2-1*, *RH2-2*, *SWS2-A*, *SWS2-B*, and *SWS1*, and the housekeeping genes beta actin (*ACTB*), cytochrome c oxidase subunit I (*COI*), and glyceraldehyde 3-phosphate dehydrogenase (*GAPDH*) were performed using a StepOnePlus™ Real-Time PCR System (Thermo Fisher Scientific). Each 20-μL reaction mixture contained 10 μL of Power SYBR® Green PCR Master Mix (Thermo Fisher Scientific), 0.4 μM of gene specific primer pairs, and 2 μL of 10-fold diluted cDNA samples. Reactions were performed with 1 cycle at 95 °C (10 min), followed by 45 cycles of 95 °C for 15 s and 60 °C for 1 min, followed by melting-curve analyses (initial temperature 60 °C, increasing by 0.3 °C/sec). Primers were designed to amplify short (80–250 bp) fragments for opsin and reference genes, and were synthesized by either Eurofins Genomics or Nihon Gene Research Laboratories. Separate qPCR assays on individual eyes were run in parallel and replicated three times on separate plates (i.e., conducting three technical replicates for each individual). Transcript copy numbers of nine cone opsin genes and three housekeeping genes were calculated using a standard curve method with plasmid standards containing each transcript. The average values of three replicates were used. We used two measures for evaluating gene expression values described by Fuller and Claricoates [51]; 1) the expression values for each opsin gene relative to geometric mean expression of the three housekeeping genes and; 2) the proportional expression of each opsin relative to the total sum of expression of nine cone opsin genes. All the primer sequences for qPCR assays and plasmid standard values are listed in Additional file 1: Table S3.

### Determination of LWS-1 opsin genotypes

*LWS-1* genotypes were determined to investigate the effects of allelic spectral variations in LWS-1 opsin on behavioral sensitivities to long wavelengths of light. Genomic DNA was extracted from caudal fins using Wizard® Genomic DNA Purification kits (Promega), and PCR primers were designed to target 5′ and 3′ UTR regions of *LWS-1* according to published sequences of Cumana [30] and Trinidadian [31] guppy opsin genes. Complete coding sequences of *LWS-1* genes were then amplified and sequences were determined using an ABI3130 Genetic Analyzer system (Thermo Fisher Scientific). *LWS-1* genotypes were determined according to Ala/Ser polymorphism at 180th amino acid residues, which are known to affect absorption spectra of opsins (A-type LWS-1, AB748984; S-type LWS-1, AB748985), as indicated by Tezuka et al. 2014 [31]. Genotypes are defined as AA180 (homozygous for the A-type allele), AS180 (heterozygous), and SS180 (homozygous for the S-type allele). Primers for PCR and sequencing are listed in Additional file 1: Table S4.

### Data analyses

All analyses were performed using the R statistical package, version 3.1.2 [52]. The effects of the light environment during growth and the stimulus light wavelength used in optomotor experiments on behavioral sensitivities to the stimulus wavelengths were analyzed using generalized linear mixed models (GLMMs) with individuals included as a random effect. We considered the light environment during growth, light wavelengths used in optomotor experiments, and their interactions as fixed effects. The effects of light environment during growth on the expression levels of opsin genes relative to housekeeping genes were analyzed using GLMMs for each cone opsin gene, with light environment during growth as a fixed effect. We did not analyze the effect of sex because the number of individuals of both sexes was insufficient. We examined the effects of opsin gene expression on behavioral sensitivities to the stimulus wavelengths using the proportional expression values of cone opsin genes. The proportional expression values of several cone opsin genes were negatively or positively correlated with each other (Additional file 1: Figure S4); thus, we performed PCA to obtain the composite variables of gene expression levels by summarizing these values to the PCA axes. Subsequently, we examined the effects of each PCA axis on behavioral sensitivities to four wavelengths of light using GLMs.

## Availability of data and materials

All datasets supporting the conclusions of this article are available on the Dryad Digital doi:10.5061/dryad.4qd60.

## Ethics

All procedures for animal care and breeding were performed according to the guidelines of the animal care and use committee of Tohoku University.

## Additional file

Additional file 1: Table S1. Proportion of variance and eigenvalues for the first three principal components from opsin gene expression (proportional expression) data. **Table S2.** The mean (± SEM) lens density values at which optomotor responses were less than 0.5 (gain threshold). **Table S3.** Primers for real-time qPCR; Mean PCR efficiencies (± SE) and r-square values for each gene assay were calculated using serial dilutions of plasmid standards. **Table S4.** PCR and sequencing primers for *LWS-1.* **Figure S1.** Characteristics of green and orange experimental light environments; (A) Relative irradiance spectra of green and orange light environments during growth; spectra were normalized to peak intensity values; (B) Computed quantum catches by visual opsins of green and orange light spectra. **Figure S2.** The attenuation of light intensity (photon flux density) of the four stimulus wavelengths by neutral density filters. Downwelling light intensities of stimulus light were measured in the middle of the acrylic cylinder tank using a spectrometer (USB2000). **Figure S3.** Computed quantum catches by visual opsins of 532, 546, 570 and 600-nm light. **Figure S4.** Cross-correlation matrix of gene expression of cone opsins relative to house keeping genes. Correlation coefficients among cone opsin gene expression values were displayed by heat map. In the color spectrum bar, red and blue indicate positive and negative correlation, whereas gray indicates no correlation. (DOCX 265 kb)
