# Supplementary material for: Effects of light environment during growth on the expression of cone opsin genes and behavioral spectral sensitivities in guppies (Poecilia reticulata)
Source: BMC Evol Biol. 2016 May 18;16:106. doi: 10.1186/s12862-016-0679-z (PMC4870739; doi:10.1186/s12862-016-0679-z)
Supplement: Additional file 1: Table S1. — Proportion of variance and eigenvalues for the first three principal components from opsin gene expression (proportional expression) data. Table S2. The mean (± SEM) lens density values at which optomotor responses were less than 0.5 (gain threshold). Table S3. Primers for real-time qPCR; Mean PCR efficiencies (± SE) and r-square values for each gene assay were calculated using serial dilutions of plasmid standards. Table S4. PCR and sequencing primers for LWS-1. Figure S1. Characteristics of green and orange experimental light environments; (A) Relative irradiance spectra of green and orange light environments during growth; spectra were normalized to peak intensity values; (B) Computed quantum catches by visual opsins of green and orange light spectra. Figure S2. The attenuation of light intensity (photon flux density) of the four stimulus wavelengths by neutral density filters. Downwelling light intensities of stimulus light were measured in the middle of the acrylic cylinder tank using a spectrometer (USB2000). Figure S3. Computed quantum catches by visual opsins of 532, 546, 570 and 600-nm light. Figure S4. Cross-correlation matrix of gene expression of cone opsins relative to house keeping genes. Correlation coefficients among cone opsin gene expression values were displayed by heat map. In the color spectrum bar, red and blue indicate positive and negative correlation, whereas gray indicates no correlation. (DOCX 265 kb) [file 12862_2016_679_MOESM1_ESM.docx]

**Table S1.** Proportion of variance and eigenvalues for the first three principal components from opsin gene expression (proportional expression) data

|  | PC1 | PC2 | PC3 |
| --- | --- | --- | --- |
| Proportion of Variance | 0.37 | 0.23 | 0.19 |
| *LWS-1* | 0.11 | 0.55 | 0.37 |
| *LWS-2* | 0.10 | 0.11 | −0.63 |
| *LWS-3* | −0.36 | 0.31 | 0.24 |
| *LWS-4* | −0.42 | 0.08 | −0.25 |
| *RH2-1* | 0.34 | −0.52 | −0.07 |
| *RH2-2* | −0.39 | 0.07 | −0.42 |
| *SWS2-A* | −0.48 | −0.21 | −0.00 |
| *SWS2-B* | 0.17 | 0.43 | −0.33 |
| *SWS1* | −0.39 | −0.26 | 0.21 |

**Table S2.** The mean (± SEM) lens density values at which optomotor responses were less than 0.5 (gain threshold).

|  | 532 nm | 546 nm | 570 nm | 600 nm |
| --- | --- | --- | --- | --- |
| Green | 3.42 ± 0.015 | 3.00 ± 0.049 | 3.03 ± 0.041 | 2.58 ± 0.051 |
| Orange | 3.36 ± 0.013 | 3.21 ± 0.038 | 3.21 ± 0.038 | 3.06 ± 0.042 |

**Table S3.** Primers for real-time qPCR; Mean PCR efficiencies (± SE) and r-square values for each gene assay were calculated using serial dilutions of plasmid standards.

| Gene | Forward Primer | Reverse Primer | Product Size | PCR Efficiency [%] | R^2^ value |
| --- | --- | --- | --- | --- | --- |
| *LWS-1* | AAACAGGTGGATGATGGTTCT | CAATAAATAGTTTCTGTACAGGTCAC | 121 | 98.0 ± 0.39 | 1.00 |
| *LWS-2* | TGCAGCAGCTAGATAGTGAATCAA | AAAGCAGGCGAAAGTGGCA | 119 | 99.1 ± 0.74 | 1.00 |
| *LWS-3* | CACCATCTACAACCCTGTTAT | CTGTGCAGGTGACAGTAGTTTA | 194 | 90.9 ± 0.45 | 1.00 |
| *LWS-4* | ACCCAGAACGCCCAGAAG | GCCGTCATCCACCTCTTTC | 258 | 96.6 ± 0.70 | 1.00 |
| *RH2-1* | GCCTCGTCATGACAGTCAAA | AAGCCCAACACCATCAAGAC | 109 | 94.3 ± 0.32 | 1.00 |
| *RH2-2* | TGGCCATTCCTGCCTTTTTC | TCCAATTGTTGCCAGCATGC | 86 | 101.3 ± 0.32 | 1.00 |
| *SWS2-A* | GATGGAGCAGGTACATCCCA | CTGGTCACCTCCTTCTCAGC | 235 | 85.2 ± 0.46 | 1.00 |
| *SWS2-B* | TCGGGAACTTTGCCTTCAAG | TCCGCATGAACACTGCATTC | 137 | 98.7 ± 0.78 | 1.00 |
| *SWS1* | GAGCTCCTGCGTCTACAACC | TTCCCAAACACCATTTCCAT | 84 | 94.9 ± 0.40 | 1.00 |
| *COI* | GGGACTTACGGGAATTGTTCTA | GTGCTGTGAAGTGTGTAACCTG | 171 | 96.0 ± 0.20 | 1.00 |
| *β-actin* | GTGCTGTCTTCCCATCCATC | GCTCTGGGCTTCATCACCTA | 98 | 90.5 ± 0.41 | 1.00 |
| *GAPDH* | ATCCATTCATCGACCTGGAG | GACCAGCTTGTCTCCCTCTG | 101 | 96.1 ± 0.49 | 1.00 |

**Table S4.** PCR and sequencing primers for *LWS-1*

|  | Direction | Primer Sequence |
| --- | --- | --- |
| PCR primer F | Forward | TGGCACACGGTGTCTGTAAT |
| PCR primer R | Reverse | ACAGGTCACAGGTCCAAAACA |
| Sequencing Primer | Reverse | GATGGCCAACCACACAGCC |

**Figure S1.** Characteristics of green and orange experimental light environments; (A) Relative irradiance spectra of green and orange light environments during growth; spectra were normalized to peak intensity values; (B) Computed quantum catches by visual opsins of green and orange light spectra.


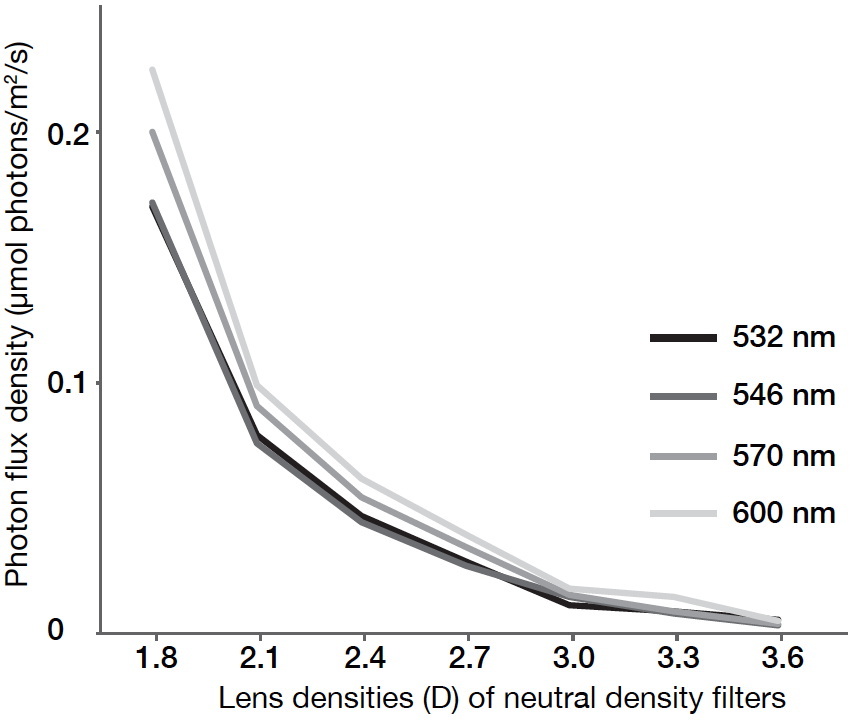


**Figure S2.** The attenuation of light intensity (photon flux density) of the four stimulus wavelengths by neutral density filters. Downwelling light intensities of stimulus light were measured in the middle of the acrylic cylinder tank using a spectrometer (USB2000).

**Figure S3.** Computed quantum catches by visual opsins of 532, 546, 570 and 600-nm light.

**Figure S4.** Cross-correlation matrix of gene expression of cone opsins relative to house keeping genes. Correlation coefficients among cone opsin gene expression values were displayed by heat map. In the color spectrum bar, red and blue indicate positive and negative correlation, whereas gray indicates no correlation.
